# Application of a Global Multiparameter Scoring System for the Prenatal Prediction of Coarctation of the Aorta

**DOI:** 10.3390/jcm10163690

**Published:** 2021-08-20

**Authors:** Enery Gómez-Montes, Ignacio Herraiz García, David Escribano Abad, Jesús Rodríguez Calvo, Cecilia Villalaín González, Alberto Galindo Izquierdo

**Affiliations:** Fetal Medicine Unit, Department of Obstetrics and Gynecology, University Hospital 12 de Octubre, 12 de Octubre Research Institute (imas12), Complutense University of Madrid, 28041 Madrid, Spain; mariaaenery.gomez@salud.madrid.org (E.G.-M.); ignacio.herraiz@salud.madrid.org (I.H.G.); d_escribano@yahoo.es (D.E.A.); jesusrodriguezcalvo@yahoo.es (J.R.C.); ceci.gvillalain@gmail.com (C.V.G.)

**Keywords:** congenital heart disease, fetal echocardiography, coarctation of the aorta, prediction, z-score, multiparameter score

## Abstract

To assess prospectively the capability of our previously reported global multiparameter scoring system to predict coarctation of the aorta (CoAo) in fetuses with cardiac asymmetry, we applied and analyzed the performance of our scoring system in predicting postnatal CoAo in fetuses undergoing prenatal echocardiographic assessment because of cardiac asymmetry between 2011 and 2021, and we determined the cut-off points of the score with the best balance between specificity and sensitivity, and of maximum sensitivity and specificity. CoAo was confirmed in 39/179 newborns (21.8%). We found a significantly higher probability of CoAo in fetuses with CoAo than in cases without CoAo (84.2 ± 18.2% vs. 26.0 ± 28.6%, *p* < 0.001). The AUC of the ROC of the score was 0.93 (95% CI 0.89–0.97). The cut-off value with the best balance between specificity and sensitivity was a predicted risk of ≥53% (sensitivity 92.3% and specificity 80.0%). The cut-off point of maximum sensitivity was ≥35% (sensitivity 100% and specificity 72.9%), and that of maximum specificity was ≥96% (sensitivity 43.6% and specificity 96.4%). In none of the fetuses with a probability of CoAo < 35% was this condition confirmed after birth. This occurred in 102 fetuses in the whole study population (57%) and in 84 of the 111 in whom CoAo was suspected beyond 28 weeks (75.7%). This multiparameter score allows an adequate discrimination between fetuses without CoAo and those with CoAo, reducing the false positive diagnoses in cardiac asymmetry.

## 1. Introduction

Coarctation of the aorta (CoAo) is one of the most common congenital heart defects (CHD) [1], accounting for about 7% of all live births with CHD [2]. CoAo is also the most frequently undiagnosed critical cardiovascular disease during the early neonatal period [3], especially if isolated, it being diagnosed after discharge in 60% of cases [3,4]. The prenatal detection rate of this CHD is undesirably low [5,6], having a high false positive rate because its prenatal diagnosis depends on unspecific and indirect findings (cardiac asymmetry with right dominance) [1,7,8,9,10,11,12]. This cardiac asymmetry is based on the evaluation of the four chambers and outflow tract planes, such that the left ventricle and/or ascending aorta are smaller than the right ventricle and pulmonary artery, respectively, and its predictive capability depends on the gestational age (GA) of onset. The positive predictive value of this cardiac asymmetry varies from 60% to 86% in the second trimester and decreases to 10–41% in the third trimester [1,7,11,13,14], because this finding could appear in fetuses without CoAo in late pregnancy [15]. Consequently, more than 50–60% of cases with a prenatal suspected CoAo are not confirmed after birth, but they are recommended to be delivered at tertiary hospitals [7,16,17,18,19,20,21,22,23,24,25,26]. An improvement in survival, morbidity, and perioperative conditions when the CoAo is prenatally diagnosed by enabling planned delivery in an adequate center and the early prevention of a ductus arteriosus (DA) constriction has been reported [27,28]. For all these reasons, distinction between those fetuses in which CoAo will be postnatally confirmed and those without CoAo is a very important issue. Previous studies have described some morphologic and functional cardiac parameters that could be useful to obtain a better prenatal prediction of CoAo [21,29,30,31]. In addition, over the last few years, several multiparameter scoring systems to differentiate between subjects with and without CoAo have been published [18,19,20,21,22,23,24,25,30,32,33]. However, there are few groups that have applied these scores to clinical practice to distinguish fetuses with and without CoAo. Our group was one of the first to report a global multiparameter scoring system based on echocardiographic parameters and the GA at diagnosis to improve risk stratification of truly having CoAo in fetuses with cardiac asymmetry [13].

The objective of this study was to evaluate the capability of this scoring system to predict CoAo in a new cohort of fetuses with isolated ventricular and great vessel disproportion with right dominance, and to calibrate the cut-off points of the score with the best balance between specificity and sensitivity, and of the maximum sensitivity and specificity in order to improve prenatal counseling and perinatal management.

## 2. Materials and Methods

We applied our global multiparameter scoring system to fetuses undergoing prenatal echocardiography at a tertiary referral hospital because of cardiac asymmetry with right dominance during the period of 2011–2021 to calculate the probability of postnatal confirmation of CoAo in each case. Although data collection was retrospective, in clinical practice, our score was systematically applied in all cases and, hence, there was no data loss. We applied the same inclusion and exclusion criteria used to design our scoring system [13,14]. Therefore, the reason for echocardiographic evaluation was the subjective recognition of cardiac asymmetry on the four chamber and outflow tracts planes, so that the left ventricle and/or the ascending aorta were smaller than the right ventricle and pulmonary artery, respectively. Only fetuses with appropriate growth without chromosomal abnormalities in which a simple CoAo was suspected and with full postnatal follow-up were included in the analysis. Therefore, we did not consider cases with associated cardiac defects. We did not exclude patients with extracardiac anomalies that did not interfere with the standard postnatal management of CoAo; that is, those conditions that did not require surgical correction during the neonatal period or, if this was required, did not imply a change in the postnatal echocardiographic follow-up or in the surgical repair of CoAo. Moreover, we excluded those cases with extracardiac anomalies that can produce cardiac asymmetry because of an extrinsic compression, such as congenital diaphragmatic hernia or congenital cystic adenomatoid malformation with significant mediastinal shift. The study was approved by our hospital’s ethics committee.

The echocardiographic study protocol applied to fetuses with cardiac asymmetry did not change with respect to what we employed in these cases before performing the score, and in the same way, our perinatal management did not change depending on the result of the score. Fetal examinations involved an exhaustive extracardiac structural examination and a complete echocardiography [34,35]. The approach for echocardiographic measurements has been detailed elsewhere [13]. Briefly, cardiac measurements were taken at their maximum size (inner edge to inner edge). The diameter of the aortic isthmus was measured immediately proximal to the insertion of the DA in the sagittal and in the three vessels and trachea (3VT) views, and the DA diameter was measured only in the 3VT view. At least three measurements of each dimension were taken from different frames and the mean was used. These dimensions and the ratio between the right and left cardiac structures (tricuspid valve (TV)/mitral valve (MV), pulmonary valve (PV)/aortic valve (AV), and main pulmonary artery (MPA)/ascending aorta (AAo)) were taken during diagnostic echocardiography. The Z-scores for cardiac measurements were calculated, employing appropriate software [36,37,38]. Following the same methodology of our original study [13], the Z-scores were calculated according to the gestational age, which was determined based on the first trimester ultrasound, and if this reference was not available, the Z-scores were calculated based on the femoral length measured at the time of fetal echocardiography. In the current study, all cases included had a first trimester ultrasound, performed between 11 + 0 and 13 + 6 weeks, and we verified in all of them that the gestational dating was correct, following our protocol [39]. According to this, we employed the last menstrual period (LMP) for gestational dating as long as it is reliable; that is, pregnant patients had to remember with certainty the first day of the LPM, menstrual cycles needed to be regular (24–32 days), and they must not have used oral contraceptives or breastfed for two months prior to the LPM. If any of these conditions was not fulfilled, we assessed the gestational age based on the measurement of the fetal crown-rump length. However, even if LMP is reliable, whenever there is a discordance of more than 7 days between the gestational age dating based on LMP and the ultrasound, the fetal crown-rump length was used to establish the gestational age. Therefore, in this study, the reference for calculating the Z-score values was always the same, namely gestational age. We also recorded the subjective assessment of the appearance of the aortic arch (diffuse hypoplasia of the entire aortic arch from the ascending aorta to the isthmus or not, based on the impression of a narrowing of the whole aortic arch towards the isthmus), the persistence of the left superior vena cava (PLSVC), the presence of a infolding of the posterior wall of the aortic arch after the origin of the left subclavian artery (contraductal shelf), and the appearance of the foramen ovale (FO) (redundant or not, considered as redundant when it prolapses into the left atrium for more than 50% of the left atrial width, assessed subjectively) [40]. Additionally, functional parameters were registered including transaortic peak velocity and blood flow evaluation at the aortic arch with color Doppler (antegrade filling, mixed pattern with antegrade and retrograde ductal-dependent flows or reversed filling from the ductus) and FO (right-to-left, left-to-right or bidirectional). We defined ‘mixed’ flow at the aortic arch when both features, antegrade and reversed ductal-dependent flow, contribute approximately proportionately to its filling. Flow at the FO was described as ‘bidirectional’ when the volume of left-to-right shunting was the same or even higher than right-to-left shunt. 

The score we applied was composed of GA at diagnosis, Z-score of the AAo, Z-score of the aortic isthmus in the 3VT view and PV/AV ratio. It was applied in the first echocardiographic assessment performed for suspected CoAo. At that time, the probability of CoAo was established using the pre-test odds for CoAo obtained in our previous study and the likelihood ratios resulting from each parameter (Table 1). All the cardiac parameters as well as the ratio between right and left cardiac structures (TV/MV, PV/AV, and MPA/AAo) and the probability of CoAo prenatally evaluated by the global score were compared between fetuses with and without postnatal confirmation of CoAo.

Prenatal invasive testing was offered whenever high-risk clinical factors or extracardiac anomalies coexisted with suspected CoAo. In all other cases, the karyotype was considered postnatally according to clinical findings. Ultrasound exams were carried out by fetal medicine specialists, employing high-quality equipment with Doppler and B-mode adjustments for fetal heart examination. All cases were evaluated together with a pediatric cardiologist. Subsequent ultrasound examinations every 4–6 weeks were recommended in ongoing pregnancies, as well as perinatal management in an adequate tertiary center.

Given the close relationship detected between GA at initial diagnosis and the probability of CoAo, we considered two subgroups of interest for additional analysis: the group with early-onset cardiac asymmetry, diagnosed at ≤28 weeks; and the group with late-onset cardiac asymmetry, diagnosed at >28 weeks. This GA cut-off point was chosen according to the results of our original study in which this cut-off value had the best predictive performance [13].

Postnatal outcome was determined from our own hospital’s medical records (Table S1). We requested any additional relevant information from the referring hospital if necessary. The postnatal management of the CoAo has been detailed elsewhere and it was consistent during the study period [13]. Patients in which CoAo was not confirmed postnatally underwent a cardiologic follow-up consisting of an echocardiography at 1 and 6 months after birth in order to diagnose late CoAo [41]. All newborns with prenatally suspected CoAo underwent an echocardiogram in the first 2 h after birth. At least a 6-month postnatal follow-up was available for all surviving cases.

### Statistical Analysis

Data were expressed in mean ± standard deviation or count (%) unless otherwise specified. Comparisons were determined for all cardiac parameters based on postnatal outcome (confirmed CoAo vs. false positive). Continuous variables were explored by *t*-test or Mann–Whitney test, and categorical variables by chi-square or Fisher exact test, where appropriate. We assessed the predictive capability of our global multiparameter scoring system by analyzing the area under the ROC curve (AUC), and we established the cut-off points with the best balance between specificity and sensitivity, and of maximum sensitivity and specificity. We considered *p* < 0.05 as the criterion for statistical significance, and *p*-values were two-sided for all tests. Data were analyzed by applying SPSS 20.0 (SPSS, Chicago, IL, USA).

## 3. Results

**Figure 1:**
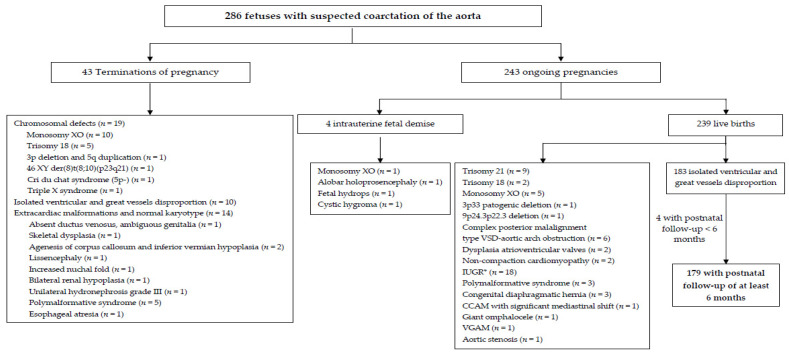
Flow chart of fetuses with cardiac asymmetry according to the selection criteria. CCAM, congenital cystic adenomatoid malformation; IUGR, intrauterine growth restriction; VGAM, vein of Galen aneurysmal malformation; VSD, ventricular septal defect. * Diagnosis of IUGR was made before suspected coarctation of the aorta.

During the study period, in 286 fetuses, a suspicion of CoAo was established. Fetal karyotyping was carried out in 113 cases (39.5%), 38 of which had a chromosomal abnormality (38/113, 33.6%). One hundred and seven of the 286 fetuses were excluded from the complete analysis. Figure 1 shows the reasons for exclusion and the outcome of the whole group.

The whole study group included 179 liveborn fetuses without chromosomal abnormalities. Their most important features are summarized in Table 2. Most cases (131/179, 73.2%) were diagnosed in the background of routine ultrasound examinations carried out in either the second or third trimester, and in the remaining cases (48/179, 26.8%), the diagnosis was made at unscheduled scans (three cases during ultrasound scans at ≤18 weeks, 26 cases between 23 and 31 weeks, and 19 cases ≥37 weeks). 

CoAo was confirmed postnatally in 39 newborns (21.8%), and in the remaining 140 it was excluded. Most patients (35/39, 89.7%) underwent surgical correction, and in the remaining four, an expectant management was chosen. Surgical correction was carried out at a mean age of 14.7 ± 19.4 days (median 8.0 days, range 2–88). All cases are now alive and well, except for one of them, who died 3 weeks after surgery because of fungal mediastinitis. Six patients (6/35, 17.1%) required surgical repair for re-coarctation at a mean age of 106.3 ± 50.2 days (median 102.5 (range, 31–175 days)). The mean postnatal follow-up time was 58.9 ± 33.7 months (median 55.6 months, range 6.5–118.3 months).

### 3.1. Prenatal Prediction of CoAo at First Diagnostic Echocardiography

The ultrasound characteristics at the first prenatal diagnostic echocardiographic scan based on the postnatal outcome (CoAo vs. no CoAo) are available in the supplementary material section (Table S1). Fetuses with confirmed CoAo were detected significantly earlier and had significantly lower Z-scores for the MV, AV, AAo, and aortic isthmus, in both the 3VT and sagittal planes. Likewise, the TV/MV, PV/AV and MPA/AAo ratios were larger and isthmus/arterial duct ratio was smaller in cases with CoAo. There were also more cases of diffuse hypoplasia of the aortic arch in CoAo fetuses. However, we did not find significant differences between the postnatal outcome groups regarding functional features, PLSVC or the size of right cardiac structures. In most cases, the blood flow at the aortic arch was antegrade (165/179, 92.2%), in five cases it was reversed (5/179, 2.8%), and in nine it had a mixed pattern (antegrade and reversed) (9/179, 5.0%). In all cases, the flow at the FO was normal (right to left), except in one case, in which was left to right. 

**Figure 2:**
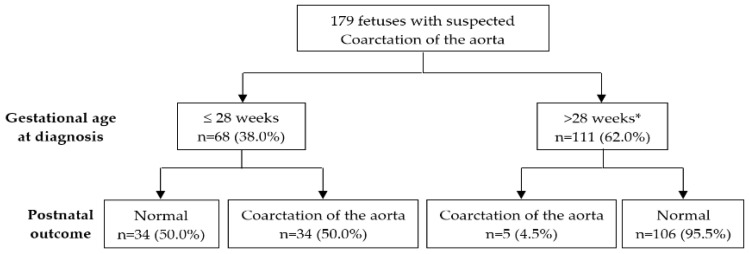
Postnatal outcome based on the gestational age at diagnosis. * All fetuses underwent ultrasound scan at mid-second trimester (19–22 weeks) which was informed as normal, except for three cases in which this scan was not performed, and in all 3 of these cases, CoAo was not postnatally confirmed.

The definitive postnatal outcomes according to the gestational age of initial prenatal diagnosis are summarized in Figure 2.

No cases of contraductal shelf were observed in our study group. However, redundant foramen ovale flap (RFOP) was found more often in fetuses without CoAo. There were 30 cases of RFOP, of which 25 (25/30, 83.3%) occurred in late-onset cardiac asymmetries, and the remaining 5 (5/30, 16.7%) in early-onset cases (*p* = 0.008). CoAo was postnatally confirmed in only one case, which belonged to the early-onset group.

### 3.2. Prospective Application of the Global Scoring System

**Figure 3:**
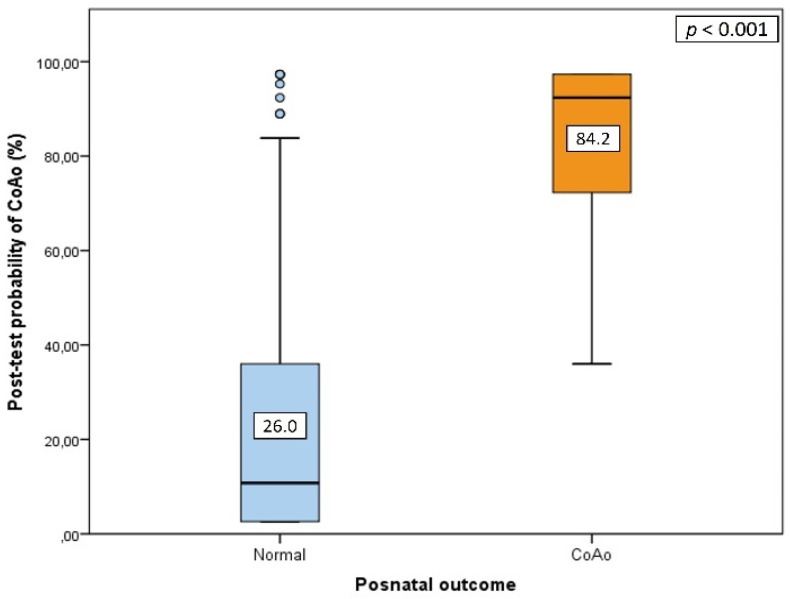
Box-and-whisker plot illustrating the distribution of post-test probability of coarctation of the aorta (CoAo) according to postnatal outcome by applying our global multiparameter score. Solid horizontal lines inside boxes correspond to median values, while the interquartile range (IQR) is represented by boxes. Whiskers show range. Values beyond 1.5 IQR from 25th and 75th centiles are shown by extreme points. Numbers in boxes indicate mean values.

After applying the global score to the study group and calculating the post-test probability of CoAo according to the described method (Table 1), we found a significantly higher probability of CoAo in fetuses with a postnatal confirmation of CoAo than in cases without CoAo (84.2% ± 18.2% vs. 26.0% ± 28.6%, *p* < 0.001) (Figure 3). 

**Figure 4:**
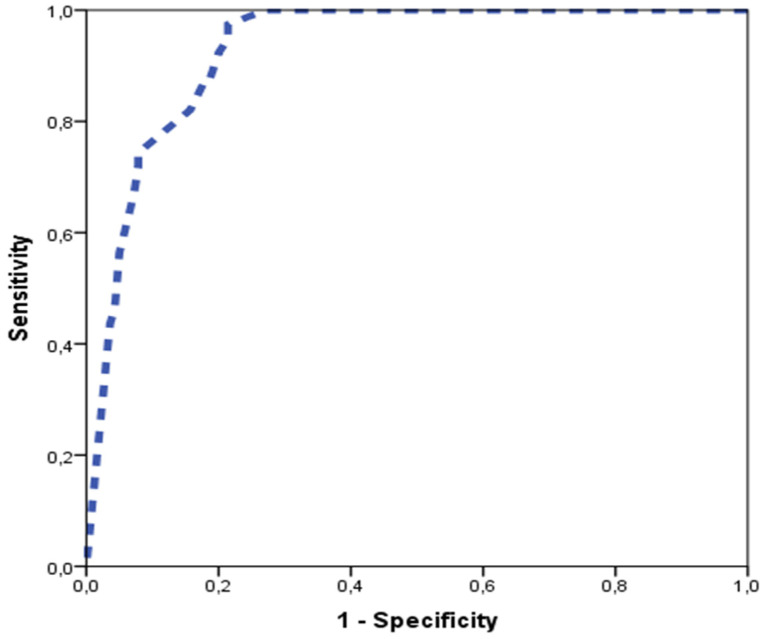
Receiver operating characteristics curve of the global multiparameter score prospectively applied (area under the curve (AUC), 0.93 (CI 95% 0.89–0.97)).

The AUC of the ROC curve of the global score was 0.93 (95% CI 0.89–0.97) (Figure 4). The predictive performance of each single parameter that composes the score (GA at diagnosis, Z-score of the AAo, Z-score of the aortic isthmus in the 3VT view, and PV/AV ratio), calculated by analyzing its ROC curve and the AUC, was: 0.86 (95% CI 0.79–0.92), 0.86 (95% CI 0.81–0.92), 0.80 (95% CI 0.74–0.87), and 0.79 (95% CI 0.72–0.87), respectively.

**Figure 5:**
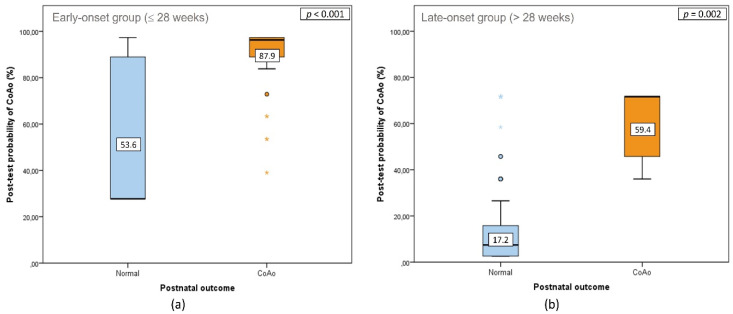
Box-and-whisker plots illustrating the distribution of post-test probability in 68 fetuses with suspected coarctation of the aorta (CoAo) diagnosed at ≤28 weeks (**a**) and 111 at >28 weeks (**b**), according to postnatal outcome. Solid horizontal lines inside boxes correspond to median values, while the interquartile range (IQR) is represented by boxes. Whiskers show range. Values beyond 1.5 IQR from 25th and 75th centiles are shown by extreme points. Numbers in boxes indicate mean values.

Additionally, we compared the performance of the multiparameter scoring system in early and late-onset subgroups. The resulting estimated probabilities of postnatal confirmation of CoAo are shown in Figure 5. As expected, in the early diagnosis group, there was a mean probability of CoAo that was significantly higher in CoAo fetuses than in normal fetuses (87.9 ± 15.4% vs. 53.6 ± 30.0, *p* < 0.001). In the same way, in the late diagnosis group, a mean probability of CoAo that was significantly higher was found in CoAo fetuses than in fetuses without CoAo (59.4 ± 17.2% vs. 17.2 ± 21.8%, *p* = 0.002). The AUC of the global score applied in the early group was of 0.82 (95% CI, 0.71–0.92) and in the late group of 0.91 (95% CI, 0.85–0.98). Five cases in the early-onset group with CoAo had an extremely low probability of CoAo (orange asterisks in Figure 5a). These cases had no other associated findings, and no anomalies were found after birth. Similarly, five cases in the late-onset group without CoAo had an extremely high probability of CoAo (blue asterisks in Figure 5b). These cases had a Z-score of the aortic isthmus in the 3VT ≤ −2.5, with no other associated findings except for a RFOP in two fetuses. Postnatal evaluation did not reveal any anomalies. 

From the ROC curve of the global score, we selected the cut-off point with the best balance between specificity and sensitivity, and the cut-off values of maximum sensitivity and specificity for the diagnosis of CoAo in the whole study population and in the early- and late-onset cardiac asymmetry subgroups (Table 3).

The cut-off point with the best balance between specificity and sensitivity and the cut-off value of maximum specificity differ among the groups (whole population, early-onset, and late-onset), with higher cut-off points in the early-onset group and lower cut-off values in the late-onset group. However, the cut-off point of maximum sensitivity is similar between these groups, and as a result, a probability of postnatal confirmation of CoAo of 35% would be valid in all groups as the cut-off value of maximum sensitivity. There were 102 fetuses with a probability of postnatal confirmation of CoAo < 35%, and in none was CoAo confirmed after birth. This accounts for 57.0% of the whole study population (102/179), 26.5% of the early diagnosis group (18/68) and 75.7% of the late diagnosis group (84/111). A probability of postnatal confirmation of CoAo between 35% and 95% was found in 53 cases (53/179, 29.6%), of which in 32 patients (32/53, 60.4%) CoAo was confirmed after birth. Finally, in 24 fetuses, the probability of postnatal confirmation of CoAo was ≥96% (24/179, 13.4%), and in 18 of them (75.0%), CoAo was postnatal confirmed.

## 4. Discussion

Recent studies have proposed some morphological and functional fetal echocardiographic parameters, and even multiparameter scoring systems in order to improve the prenatal prediction of the likelihood of postnatal confirmation of CoAo [18,19,20,21,22,23,24,25,30,31,32,33]. The current study is the largest to prospectively assess the capability of a multiparameter scoring system to predict CoAo in fetuses with ventricular and great vessel disproportion with right dominance. This study shows that the prospective application of our global multiparameter scoring system to fetuses with cardiac asymmetry with right dominance allows an adequate discrimination between fetuses with and without CoAo. This is achieved both if the asymmetry appears early (≤28 weeks), as well as late (>28 weeks) in gestation, with the combination of several echocardiographic parameters, compared to the isolated assessment of a single one, being more useful at the time of the prenatal diagnosis of CoAo. It is of note that in fetuses with cardiac asymmetry and a probability of postnatal confirmation of CoAo < 35%, parents can be comforted that it is extremely unlikely that their baby has CoAo. On the other hand, in those with a probability of postnatal confirmation of CoAo ≥ 96%, it is most likely that, after birth, CoAo will be confirmed. Therefore, this global multiparameter scoring system may reduce unnecessary testing and prenatal transfers, and may lessen parental anxiety. This is of paramount value in suspected CoAo, a CHD whose prenatal diagnosis is based mainly on the finding of cardiac asymmetry, with reports proving high false positive rates associated with this indirect sign. According to our results, referral and admission to a tertiary center of more than half (57.0%) of the cases with prenatal suspected CoAo could be avoided, and, specifically, in more than three quarter of fetuses (75.7%) with late-onset cardiac asymmetry (>28 weeks), which are the most frequent cases (almost two thirds of all prenatal cardiac asymmetries were of late-onset). This has critical impact for parental counseling, postnatal care planning, and decision making [1,7,11,13,29]. 

Table 4 summarizes the echocardiographic variables and multiparameter scores proposed by several groups for prenatal prediction of CoAo [18,19,20,21,22,23,24,25,30,31,32,33]. Our global score includes three cardiac parameters (Z-score of the AAo and of the aortic isthmus in the 3VT view, and the PV/AV ratio), and GA at diagnosis. These markers are easy to assess in qualified hands, are commonly included in the echocardiographic examination of fetuses with most types of CHD (especially in right and left cardiac lesions), and have good inter- and intra-observer reproducibility [29]. Furthermore, they are objective measurements and, therefore, they are less predisposed to bias than the subjective parameters, as well as being less difficult to assess than the morphology of the aortic arch with two-, three- or four-dimensional ultrasound, the assessment of the isthmus–ductal angle or the evaluation of blood flow disturbances using color Doppler sonography, which have also been reported as predictive parameters [1,19,20,21,22,24,29,30,42,43,44].

Compared to our previous study [13], in this one, the proportion of CoAo of fetuses with cardiac asymmetry is lower (21.8 % vs. 48.2%). After the publication of our global scoring system for prenatal prediction of CoAo, increased awareness of the importance of prenatal screening of CoAo has been raised and as a result, more fetuses with suspected CoAo have been referred to our unit, mainly in late pregnancy. In fact, during this study period, we dealt withalmost more than twice the cases of late-onset cardiac asymmetry per year compared to our original study. As a consequence of the lower incidence of the CoAo in this population compared to the original study, the probability of CoAo is slightly overestimated. Nevertheless, with this score, we are able to distinguish between fetuses without CoAo and those with CoAo, with cut-off points that allow determining with relative confidence the occurrence or not of CoAo.

In our series, we have found more cases of RFOP in fetuses in which CoAo was not confirmed after birth, mainly when the suspicion of CoAo had a late onset (>28 weeks). This is probably the consequence of a limitation to the entrance of blood flow to the left ventricle caused by the occupation of a large part of the left atrial cavity by the foraminal flap. This reduction in the left ventricular preload would produce a cardiac asymmetry with right dominance, simulating what occurs with CoAo, thus being a possible source of false positives for this condition. This hypothesis is similar to that recently proposed by Vena et al. [40], where they also observed that RFOP can lead to cardiac asymmetry with right dominance, mimicking what happens with CoAo. They postulated two events as the cause of this situation: a dynamic obstruction of the mitral inflow and an obstruction to blood flow coming from the ductus venosus to the right atrium and left atrium. These two events are produced by the partial obstruction from the flap of the FO and by a variation of the septum primum normal angle. Therefore, in fetuses with late-onset cardiac asymmetry in whom a low probability of CoAo (<35%) is obtained with the global score, the observation of a RFOP could further support the fact that it is most likely that CoAo will not be confirmed after birth, although this requires further investigation.

Regarding functional parameters (flow at the aortic arch and FO, peak transaortic velocity), we did not find differences between CoAo and no CoAo fetuses. In previous studies [20], it has been observed that the peak transaortic velocity is significantly greater in those fetuses in which CoAo is confirmed after birth and require surgery. In the same way, a bidirectional or left–right interatrial shunt and a bidirectional/retrograde flow at the aortic arch have been reported more frequently in fetuses with CoAo, but with a low specificity [33]. However, as in our series, other studies did not observe differences for functional parameters (flow at the aortic arch and FO) between CoAo and no CoAo fetuses [18,20,24]. We also did not find differences for PLSVC according to postanal outcome (CoAo vs. no CoAo), similarly to other groups [18,22,24,25,33]. Nevertheless, Morgan et al. [20] found more cases of PLSVC in fetuses with CoAo compared to normal cases, although in the multivariate analysis, it was not statistically significant. In a recent meta-analysis [17], the PLSVC did not reach statistical significance either.

Finally, in our series, no suggestive contraductal shelf images were described in any case, even though the assessment of this parameter is subordinate to certain subjectivity. Although its high specificity (90–98%) for the prenatal prediction of CoAo has been described, with low sensitivity (48%) [17,29,30,33], other studies have also failed as well to demonstrate the utility of the presence of a contraductal shelf for the prenatal prediction of CoAo [20,22].

We are aware of several limitations of our study, considering first the imbalance between cases with and without postnatal confirmation of CoAo. However, this reflects, on the one hand, an increase in cases referred to us for further evaluation because of late-onset cardiac asymmetry, and, on the other hand, that the indirect signs (cardiac asymmetry with right dominance) have a low predictive value for CoAo and a high false positive rate. Precisely for this reason, our multiparameter scoring system is especially useful, since it helps to discern between fetuses with cardiac asymmetry in whom a CoAo is truly underlying from those in which it is not, identifying false positive cases, that account for most of the late-onset asymmetry referrals. Second, in order to apply the same inclusion and exclusion criteria employed in our original study to obtain a homogenous sample, we did not include cases with fetal growth restriction, either with chromosomal abnormalities or with cardiac asymmetry associated with major CHD or extracardiac anomalies with a cardiac extrinsically compression. It will be necessary to evaluate the performance of our multiparameter scoring system in fetuses with these conditions.

## 5. Conclusions

In conclusion, this is the largest study to prospectively assess the capability of a multiparameter scoring system to predict CoAo in fetuses with ventricular and great vessel disproportion with right dominance. It shows that our global score allows an adequate discrimination between fetuses with and without CoAo. The objectivity and simplicity of their elements may allow its application in fetal cardiology units. This information may be useful for improving the parental counseling, in order to provide them with the most precise information concerning the outcome of their baby, as well as for planning the postnatal care, reducing unnecessary testing and prenatal transfer in more than half of cases with a prenatal diagnosis of cardiac asymmetry and right dominance.

## Figures and Tables

**Table 1:** Cut-off points and positive and negative likelihood ratios (LR+ and LR−) of parameters of our global multiparameter scoring system for calculating the probability of coarctation of the aorta (CoAo) in fetuses with cardiac asymmetry, applying the formulas described in the table [13].

Predictive Parameter	Cut-Off Value	LR + (95% CI)	LR − (95% CI)
Gestational age (weeks)	≤28	4.3 (2.0–8.8)	0.3 (0.1–0.5)
Ascending aorta Z-score	≤−1.5	2.8 (1.4–5.6)	0.4 (0.2–0.7)
Aortic isthmus (3VT) Z-score	≤−2	1.8 (0.8–3.8)	0.6 (0.3–1.2)
Pulmonary valve/aortic valve ratio	≥1.6	1.8 (1.1–3.1)	0.4 (0.2–0.9)
Pre-test odds for CoAo: 41 CoAo/44 no CoAo = 0.93 Post-test odds for CoAo: Pre-test odds × LR1 × LR2 × LR3 × LR4 Post-test probability of CoAo: Post-test odds/(Post-test odds + 1)			

CI, confidence interval; 3VT, three vessels and trachea.

**Table 2:** Prenatal characteristics of the whole study population.

Variable	Value
Whole study population, *n*	179
Mean GA at diagnosis of cardiac asymmetry ^‡^, weeks (SD, range)	29.6 (6.5, 17–39)
Detection at the 19–22 weeks’ scan *, *n* (%)	47 (26.3)
Detection at the 32–36 weeks’ scan *, *n* (%)	84 (46.9)
Mean GA at diagnosis performed at unscheduled scans, weeks (SD, range)	30.7 (6.0, 17–39)
Mean GA at the first fetal echocardiography, weeks (SD, range)	30.9 (6.2, 17–40)
Characteristics of prenatal diagnosis, *n* (%)	
Patients referred from their local centers	131 (73.2)
Suspected CHD	118
Extracardiac anomaly	6
Suspected CHD and extracardiac anomaly	3
Suspected CHD and medical history	2
Medical history	1
Others	1
Patients primarily assisting our hospital	48 (26.8)
Suspected cardiac asymmetry	43
Extracardiac anomaly	4
Suspected CHD and medical history	1
Sort of pregnancy, *n* (%)	
Single	177 (98.9)
Twin	2 (1.1)
Other minor cardiac anomalies, *n* (%)	34 (19.0)
PLSVC	26 (14.5)
VSD	5 (2.8)
ARSA	2 (1.1)
Auricular extrasystoles	1 (0.6)
Extracardiac abnormalities, *n* (%)	24 (13.4)
Single umbilical artery	7 (3.9)
Ductus venosus agenesis	1 (0.6)
Horseshoe kidney	2 (1.1)
Left isomerism	1 (0.6)
Esophageal atresia	1 (0.6)
Persistent intrahepatic right umbilical vein	1 (0.6)
Hydronephrosis	3 (1.7)
Talipes equinovarus bilateral	3 (1.7)
Cerebellar hemorrhagic stroke	1 (0.6)
Congenital cystic adenomatoid malformation	2 (1.1)
Interrupted inferior vena cava	1 (0.6)
Myelomeningocele	1 (0.6)
Mean GA at birth, weeks (SD, range)	38.7 (1.9, 27–41)
Mean birth weight, grams (SD, range)	3175.3 (576.6, 930–4300)

ARSA, aberrant right subclavian artery; CHD, congenital heart disease; GA, gestational age; PLSVC, persistent left superior vena cava; SD, standard deviation; VCI, vena cava inferior; VSD, ventricular septal defect. ^‡^ Gestational age at the ultrasound performed before the fetal echocardiographic study. * In our country, all pregnant patients have these ultrasound examinations routinely in prenatal care.

**Table 3:** Area under the curve (AUC), cut-off point with the best balance between specificity and sensitivity, and cut-off values of maximum sensitivity and specificity for the diagnosis of CoAo selected from the ROC curve of the multiparameter scoring system applied to the whole study population, and to the early- and late-onset cardiac asymmetry subgroups.

Cut-Off Point		Sn (95% CI)	Sp (95% CI)	NPV (95% CI)	PPV (95% CI)	AUC (95% CI)
Whole study population						
Best balance between Sn and Sp	≥53%	92.3 (79.7–97.3)	80.0 (72.6–85.8)	97.4 (92.6–99.1)	56.3 (44.1–67.7)	0.93 (0.89–0.97)
Maximum Sn	≥35%	100 (91.0–100)	72.9 (65.0–79.5)	100 (96.4–100)	50.6 (39.7–61.5)	
Maximum Sp	≥96%	43.6 (29.3–59.0)	96.4 (91.9–98.5)	86.0 (79.7–90.6)	77.3 (56.6–89.9)	
Early-onset cardiac asymmetry (≤28 weeks)						
Best balance between Sn and Sp	≥84%	76.5 (60.0–87.6)	70.6 (53.8–83.2)	75.0 (57.9–86.7)	72.2 (56.0–84.2)	0.82 (0.71–0.92)
Maximum Sn	≥39%	100 (89.8–100)	52.9 (36.7–68.5)	100 (82.4–100)	68.0 (54.2–79.2)	
Maximum Sp	≥96%	50.0 (34.1–65.9)	85.3 (69.9–93.6)	63.0 (48.6–75.5)	77.3 (56.6–89.9)	
Late-onset cardiac asymmetry (>28 weeks)						
Best balance between Sn and Sp	≥45%	80.0 (37.6–96.4)	86.8 (79.0–92.0)	98.9 (94.2–99.8)	22.2 (9.0–45.2)	0.91 (0.85–0.98)
Maximum Sn	≥35%	100 (56.6–100)	79.2 (70.6–85.9)	100 (95.6–100)	18.5 (8.2–36.7)	
Maximum Sp	≥71%	60.0 (23.1–88.2)	89.6 (82.4–94.1)	97.9 (92.8–99.4)	21.4 (7.6–47.6)	

CI, confidence interval; NPV, negative predictive value; PPV, positive predictive value; Sn, sensitivity; Sp, specificity.

**Table 4:** Echocardiographic variables and multiparameter scores proposed by several groups for prenatal prediction of coarctation of the aorta.

Study		Predictive Parameters/Scores	AUC (95% CI)	Sn (95% CI)	Sp (95% CI)
Jowett et al. 2012 [30] (*n* = 37)		Z-score AoIsth (3VT or sagittal) < −2 AoIsth/DA < 0.74 Contraductal shelf Continuous diastolic flow at AoIsth	0.52 (0.32–0.72)	33 (17–53)	71 (29–96)
Gómez Montes et al. 2013 [13] (*n* = 85)		GA at diagnosis ≤28 s AAo Z-score ≤ −1.5 AoIsth Z-score (3VT) ≤ −2 PV/AV ≥ 1.6	0.94 (0.87–0.99)	90 (77–96)	75 (61–85)
Gómez Montes et al. 2014 [14] (*n* = 115)	≤28 weeks	AAo Z-score ≤ −1.1 AoIsth Z-score (3VT) ≤ −1.2	0.98 (0.94–1.00)	91 (76–97)	91 (62–98)
	>28 weeks	TV/MV ≥ 1.48 MPA/AAo ≥ 1.85	0.84 (0.67–1.00)	63 (31–86)	43 (30–58)
Marginean et al. 2015 [23] (*n* = 32) Late-onset cardiac asymmetry (32–39 weeks)		RV/LV > 1.5 AoIsth/DA < 0.7 AoIsth (3VT) < 4.2 mm Three parameters present		56 (21–86)	87 (66–97)
Toole et al. 2016 [24] (*n* = 62)		MV Z-score < −1.63 MV/TV < 0.75 AoIsth/DA < 0.5I sthmus-ductal angle < 117° At least two of these parameters present	0.92 (0.80–1.00)	85 (66–96)	60 (42–76)
Anuwutnavin et al. 2016 [18] (*n* = 31)		AAs Z-score ≤ −2 (4 points) MV Z-score VM ≤ −2 (2 points) Transverse aortic arch Z-score ≤ −2 (1 point) VSD (1 point) Score ≥ 4 points		100	88
Arya et al. 2016 [21] (*n* = 40) ≥28 weeks		LCCA-LSA distance > 4.5 mm AAo-DAo angle ≤ 20.31° Transverse aortic arch-DAo angle ≥ 96.15°		95 (75–100)	100 (83–100)
Beattie et al. 2017 [22] (*n* = 62)		MPA/AAo ≤ 0.65 Diastolic flow persistence at AoIsth		87	53
Patel et al. 2018 [31] (*n* = 27)		LCCA-LSA distance (mean in CoAo 5.3 mm) DT/LCCA-LSCA index (mean in CoAo 0.59) BA-LCCA distance (mean in CoAo 3.2 mm)			
Wang et al. 2019 [19] (*n* = 69)		AoIsth Z-score (sagittal) ≤ −3.7 VTI_D_/VTI_S_ > 0.56	0.96 (0.88–0.99)		
Morgan et al. 2019 [20] (*n* = 107)		GA at diagnosis Transverse aortic arch (mm) AAo Z-scorePeak AAo Doppler velocity	0.92 (0.87–0.97)	89	82
Vigneswaran et al. 2020 [25] (*n* = 149)		AoIsth Z-score (3VT) DA Z-score			
Freeman et al. 2021 [32] (*n* = 35)		AAo-DAo angle Transverse aortic arch-DAo angle AAo			
Fricke et al. 2021 [33] (*n* = 65)		CSA index < 0.78 AoIsth/DA × MV/TV < 0.37	0.94 (0.85–1) -	92 100	97 95

AAo, ascending aorta; AoIsth, aortic isthmus; AUC, area under the curve; AV, aortic valve; BA, brachiocephalic artery; CI, confidence interval; CSA index, carotid-subclavian artery index (ratio of the aortic arch diameter at the left subclavian artery, to the distance between the left carotid artery and the left subclavian artery); DA, ductus arteriosus; DAo, descending aorta; DT, distal transverse arch diameter; GA, gestational age; LCC, left common carotid artery; LSA, left subclavian artery; LV, left ventricle; MPA, main pulmonary artery; MV, mitral valve; PV, pulmonary valve; RV, right ventricle; Sn, sensitivity; Sp, specificity; TV, tricuspid valve; VSD, ventricular septal defect; VTID, diastolic velocity-time integral at the aortic isthmus; VTIS, systolic velocity-time integral at the aortic isthmus; 3VT, three vessels and trachea.

## Data Availability

The data presented in this study are available upon reasonable request from the corresponding author. The data are not publicly available due to privacy concerns.

## Supplementary Materials

The following are available online at https://www.mdpi.com/article/10.3390/jcm10163690/s1, Table S1: Prenatal ultrasound characteristics of the whole study population based on the postnatal outcome (normal vs. coarctation of the aorta).
