# Supplementary material for: Application of a Global Multiparameter Scoring System for the Prenatal Prediction of Coarctation of the Aorta
Source: J Clin Med. 2021 Aug 20;10(16):3690. doi: 10.3390/jcm10163690 (PMC8397204; doi:10.3390/jcm10163690)
Supplement: Supplementary file 1 [file jcm-10-03690-s001.zip › jcm-1317363-supplementary.pdf]

Table S1. Prenatal ultrasound characteristics of the whole study population based on the postnatal outcome (normal vs. coarctation of the aorta).

Variable	Postnatal normal (n = 140)	Coarctation of the aorta (n = 39)	Mean difference (IC 95%)	p Value
Gestational age at diagnosis, weeks	31.6 (5.5)	22.5 (4.7)	9.1 (7.3 – 11.0)	**<0.001**
Morphologic features				
Inflow tracts				
z-score Tricuspid valve	-0.1 (0.9)	-0.2 (1.1)	0.1 (-0.2 - 0.5)	0.371
z-score Mitral valve	-1.5 (1.1)	-3.0 (1.5)	1.5 (1.0 - 2.0)	**<0.001**
Tricuspid valve/Mitral valve ratio	1.4 (0.3)	1.5 (0.3)	-0.1 (-0.2 - 0.0)	**0.041**
Outflow tracts				
z-score Pulmonary valve	0.7 (1.1)	0.8 (1.0)	-0.1 (-0.5 – 0.3)	0.568
z-score Aortic valve	-0.8 (1.2)	-2.4 (1.3)	1.6 (1.2 – 2.1)	**<0.001**
Pulmonary valve/Aortic valve ratio	1.5 (0.3)	1.8 (0.3)	-0.3 (-0.4 – -0.2)	**<0.001**
z-score Main pulmonary artery	0.9 (1.0)	0.8 (1.0)	0.1 (-0.3 – 0.4)	0.686
z-score Ascending aorta	-0.9 (1.1)	-2.5 (1.0)	1.6 (1.3 - 2.0)	**<0.001**
Main pulmonary artery/Ascending aorta ratio	1.6 (0.3)	1.9 (0.3)	-0.3 (-0.4 - -0.2)	**<0.001**
Aortic arch				
z-score Isthmus (3VT view)	-1.6 (1.5)	-3.0 (1.1)	1.4 (0.9 – 2.0)	**<0.001**
z-score Isthmus (sagittal view)	-1.6 (1.2)	-2.7 (1.2)	1.1 (0.7 – 1.6)	**<0.001**
Hypoplastic aortic arch, n (%)	15/140 (10.7)	35/39 (89.7)		**<0.001**
Others				
z-score Arterial duct	0.3 (1.2)	0.2 (1.0)	0.1 (-0.3 – 0.6)	0.588
Isthmus/Arterial duct ratio	0.7 (0.2)	0.6 (0.1)	0.1 (0.1 - 0.2)	**<0.001**
Persistent left superior vena cava, n (%)	18/140 (12.9)	8/39 (20.5)		0.230
Redundant foramen ovale flap, n (%)	29/140 (20.7)	1/39 (2.6)		**0.007**
Functional features				
Mixed-reversed flow at the aortic arch*, n (%)	9/140 (6.4)	5/39 (12.8)		0.190
Peak transaortic velocity (cm/s)	87.8 (24.0)	90.1 (26.0)	-2.3 (-17.5 – 12.7)	0.904

Quantitative results are expressed as mean (SD). Bold values are statistically significant.
3VT: 3 vessels and trachea; * Mixed pattern of flow at the aortic arch with antegrade and reversed ductal-dependent components.
